# *In vitro* Selection of Synbiotics and *in vivo* Investigation of Growth Indices, Reproduction Performance, Survival, and Ovarian *Cyp19α* Gene Expression in Zebrafish *Danio rerio*

**DOI:** 10.3389/fmicb.2021.758758

**Published:** 2021-09-04

**Authors:** Hamideh Zakariaee, Mohammad Sudagar, Seyede Sedighe Hosseini, Hamed Paknejad, Kartik Baruah

**Affiliations:** ^1^Department of Aquaculture, Faculty of Fisheries and Environmental Sciences, Gorgan University of Agriculture Sciences and Natural Resources, Gorgan, Iran; ^2^Laboratory Sciences Research Center, Golestan University of Medical Sciences, Gorgan, Iran; ^3^Department of Laboratory Sciences, Faculty of Paramedicine, Golestan University of Medical Sciences, Gorgan, Iran; ^4^Department of Animal Nutrition and Management, Aquaculture Nutraceuticals Research Group, Faculty of Veterinary Medicine and Animal Sciences, Swedish University of Agricultural Sciences, Uppsala, Sweden

**Keywords:** prebiotics, probiotics, zebrafish, mushroom, artichoke, reproduction, *cyp19a* gene

## Abstract

In this study, we tested the compatibility of two extracts from the plant Jerusalem artichokes and button mushrooms with two different *Lactobacillus* probiotics (*Lactobacillus acidophilus*; La and *Lactobacillus delbrueckii* subsp. *Bulgaricus*; Lb) to develop a synbiotic formulation to improve the growth, survival, and reproductive performances of farmed fishes. Initially, we employed *in vitro* approach to monitor the growth of the probiotic lactobacilli in the presence of the different doses of the plant-based prebiotics, with the aim of selecting interesting combination(s) for further verification under *in vivo* conditions using zebrafish as a model. Results from the *in vitro* screening assay in the broth showed that both the probiotic species showed a preference for 50% mushroom extract as a source of prebiotic. A synbiotic formulation, developed with the selected combination of *L. acidophilus*, *L. bulgaricus*, and 50% mushroom extract, showed a positive influence on the growth and reproductive performances of the zebrafish. Our findings also imply that the improvement in the reproductive indices was associated with the upregulation of a *cyp19a* gene. Overall results suggest that a combination of *L. acidophilus*, *L. bulgaricus*, and mushroom extract can be considered as a potential synbiotic for the successful production of aquaculture species.

## Introduction

The gut microbiota plays a central role in the health, well-being, growth, and disease prevention in fish (Xiong et al., 2019). The composition of the microbial communities in the fish gut is not constant and may change with nutritional status, age, rearing water, and other environmental conditions (Ringø et al., 2010; Zhang et al., 2018; Yukgehnaish et al., 2020). The microbial balance in the fish gut is crucial for its optimal metabolism and disease prevention. Over the past few years, there has been an increasing effort to steer the gut microbiota of fish toward beneficial communities as the dominance of such beneficial microbes in the fish gut has been linked to the improvement of the growth and reproductive performances, and the resistance of the fish toward pathogens (Allameh et al., 2015; Akbari Nargesi et al., 2020; Hasan and Banerjee, 2020; Kong et al., 2020; Ringø et al., 2020b; Montazeri-Parchikolaei et al., 2021). One approach to induce a healthy gut microbiome in fish has been through the administration of probiotics – live microorganisms that, when administered in adequate amounts, confer a health benefit on the host (Aydin and Şehriban, 2019; Cámara-Ruiz et al., 2020). Numerous probiotic studies have evaluated the effects of various genera, species, and strains of bacteria on the health status, disease resistance, and growth performance of farmed aquatic animals (Ashouri et al., 2018; Tan et al., 2019; Kuebutornye et al., 2020; Mohammadi Arani et al., 2021). These studies showed that the most common type of bacteria that are effective in improving the microbiota balance in the intestine of the host are the ones that belong to the *Lactobacillus* and *Bifidobacterium* genera (EFSA FEEDAP et al., 2018), although other bacteria and certain yeasts were also effective (Guluarte et al., 2019; Tarnecki et al., 2019; Banu et al., 2020). Bacteria belonging to *Lactobacillus* species have been used as probiotics in many economically important farmed fish, and it has been reported to improve the growth performance as well as to control infections caused by bacteria (such as *Aeromonas salmonicida*, *Vibrio anguillarum*, *Flavobacterium psychrophilum*, and *Edwardsiella tarda*) in fish species (Shabirah et al., 2019; Hasan and Banerjee, 2020; Kuebutornye et al., 2020; Mora-Sánchez et al., 2020; Ringø et al., 2020a). Despite all the health benefits of probiotics, a major limitation that remains for their uses as gut/health beneficial agents in farmed fish is related to the poor capacity of the administered probiotic strains to stably or even transiently colonize the host gastrointestinal mucosal surface (Suez et al., 2019; Amenyogbe et al., 2020). To overcome these limitations of probiotics, prebiotics are used together with probiotics to have a synergistic effect (an approach referred to as synbiotics) to markedly inhibit the growth of pathogenic microbes and improve the growth and/or activity of the beneficial/probiotic microorganisms in the host gut (FAO, 2007). Prebiotics are a non-viable food component that confers health benefit(s) on the host associated with modulation of the microbiota (Okolie et al., 2017; Carlson et al., 2018; Van Doan et al., 2020). The majority of studies dealing with synbiotics are conducted in humans, but over the past few years, increasing research has been focused on farmed aquatic animals (Hasan et al., 2019; Dawood et al., 2020). There is evidence to suggest that synbiotics influence the microbial ecology of the intestines of fish and play a role in causing beneficial effects on the health and growth traits of farmed fish, e.g., by preventing the negative effects imposed by infection as well as environmental stress, and by elevating the activities of the digestive enzymes, which eventually contribute to improved feed utilization and growth performances (Huynh et al., 2017; Kumar et al., 2018; Mohammadian et al., 2019; Hasyimi et al., 2020; Moustafa et al., 2020; Kong et al., 2021).

As prebiotics, the most studied in fish were inulin, mannan oligosaccharides, fructooligosaccharides, galactooligosaccharides, and nano-oligosaccharide (Cid García et al., 2020; Kishawy et al., 2020; Pietrzak et al., 2020; Zhou et al., 2020; Ghafarifarsani et al., 2021). These types of prebiotic molecules are present in a variety of plants, such as Jerusalem artichoke, cereals, leeks, asparagus, and garlic with varying degrees of polymerization, which is associated with their different functional features (Mensink et al., 2015; Bharathi et al., 2019; Harris et al., 2019; Khangwal and Shukla, 2019; Sribounoy et al., 2021). It is however important to mention that the use of food-grade prebiotic compounds derived from plant sources as a functional feed additive in the aquafeed industry is limited by the cost of the extraction process. In the interest of addressing the production cost, increasing attention has been paid to the direct use of raw plant extracts as potential sources of natural prebiotics. Jerusalem artichoke (*Helianthus tuberosus*), a natural prebiotic enriched with inulin and fructooligosaccharides, has become a focus for use as a functional feed ingredient in the diets of farmed (aquatic) animals (Tiengtam et al., 2017; Abedalhammed et al., 2020). An improvement in the growth performance, resistance toward bacterial disease, and immune responses in farmed (aquatic) animals like common carp diets (*Cyprinus carpio* L.), juvenile red tilapia (*Oreochromis* spp.), and sea cucumber as well as in poultry and swine in response to feeding Jerusalem artichoke-supplemented diet have been reported (Sewaka et al., 2019; Abedalhammed et al., 2020; Jia et al., 2020). Another plant of interest as a source of natural prebiotics is the edible mushroom owing to its richness in bioactive substances, such as functional polysaccharides, terpenes, peptides, glycoproteins, mineral elements, unsaturated fatty acids, phenolic substances, vitamin E, and vitamin C (Aida et al., 2009; Rathore et al., 2017; Safari et al., 2019; Ansari and Jadhav, 2021; Das et al., 2021). Mushrooms and their derivatives have been widely used in aquaculture for improvement of growth performance, hematological parameters, innate immunity, and diseases resistance in many cultured species (Ahmed et al., 2017; Amiri et al., 2018; Hoseinifar et al., 2019a,b; Safari et al., 2019; Rattanachan et al., 2020; Al-Maadhedy et al., 2021; Harikrishnan et al., 2021; Kumar et al., 2021). Among different cultured mushroom species, the white button mushroom, *Agaricus bisporus*, represents an interesting source of natural prebiotics because it is a safe food that is cultured worldwide (Hoseinifar et al., 2019b), and hence it is easily available for applications. Additionally, it contains a wide variety of nutraceutical substances, such as polyphenols, ergothioneine, vitamins, minerals, and polysaccharides (Tian et al., 2012; Ramos et al., 2019; Usman et al., 2021). *Agaricus bisporus* mushroom has been well-documented for its growth-promotion, immune-enhancing, and disease-resistance in some farmed fishes like grass carp (*Ctenopharyngodon idella*), common carp (*Cyprinus carpio*), catfish (*Silurus asotus*), and rainbow trout (*Oncorhynchus mykiss*) Nile tilapia (*Oreochromis niloticus*; Van Doan et al., 2017; Amiri et al., 2018; Harikrishnan et al., 2018, 2021; Hoseinifar et al., 2019b; Safari et al., 2019; Habib et al., 2021).

In this study, we tested two plant extracts (Jerusalem artichokes and button mushrooms) as potential sources of prebiotics using *in vitro* assay for their ability to facilitate the growth of two probiotic strains of interest [*Lactobacillus acidophilus* ATCC4356 (La) and *L. delbrueckii* subsp. *bulgaricus* ATCC11842 (Lb)]. Subsequently, the preferred prebiotic candidate, which showed the most prominent effect, was combined with each of the probiotic strains in an attempt to move toward identifying synbiotic combinations. The effectiveness of the synbiotic preparation was verified *in vivo* using zebrafish as a model organism by focusing on the following readouts: growth traits, survival, and reproductive performances.

## Materials and Methods

### Probiotic Bacterial Strains

Two probiotic bacterial strains, *L. acidophilus* ATCC4356 (La) and *L. delbrueckii* subsp. *bulgaricus* ATCC11842 (Lb), purchased from Persian Type Culture Collection, Iran, were used in this study. Stock cultures were prepared by mixing a pure culture of the strain, grown overnight at 37°C in De Man Rogosa Sharpe (MRS) broth medium (Merck, Darmstadt, Germany), with 25% (v/v) sterile glycerol as cryoprotectant and stored at −80°C (Haj-Mustafa et al., 2015).

### Inoculum Preparation

Working inoculums were prepared by subculturing the stock culture in MRS broth overnight at 37°C. After that, a certain volume of the subculture was transferred (1% v/v) to a fresh volume of MRS broth and incubated with shaking at 150rpm for 18h at 37°C. The bacterial suspension was then centrifuged at 8,000 x *g* for 20min (4°C), the cell pellet washed twice with sterile phosphate buffer saline (PBS), and subsequently used for the inoculation of the culture medium (Haj-Mustafa et al., 2015).

### Bacterial Growth Analysis

Growth of La and Lb was monitored by measuring the optical cell density at 600nm using a UV/Visible spectrophotometer (Shimidzo, Japan). The measured values were plotted on growth curves. The maximum specific growth rate during the exponential growth phase was calculated following the equation of Kask et al. (2003):


$$\mathrm{Ln}\;N-{\mathrm{LnN}}_{0}=\mu \;\left(t-t_{0}\right).$$

Where *t*=time, *N*=optical density at the end of the exponential growth phase (*t*), *N*_0_=optical density at the beginning of the exponential growth phase (*t*_0_), and *μ*=specific growth rate constant (h^−1^).

The doubling time was determined by the equation: T_d_=ln2/μ_max_, where, μ_max_ – maximum specific growth rate, *t*_d_- doubling time.

### Preparation of Plant Extracts as Prebiotic Sources

Two plants, Jerusalem artichokes (*H. tuberosus*; hereafter referred to as artichoke) and white button mushrooms (*A. bisporus*; hereafter referred to as mushroom), procured from a private company in Iran, were used as sources of prebiotics. The plant materials were repeatedly washed with tap water to remove the presence of any dirt and then allowed to dry until the water droplets disappeared from their surface. Each one of the artichokes and the mushroom was cut into small slices. The slices were then dipped in a 0.5% (w/v) citric acid solution for 15min to avoid browning (Mahore and Shirolkar, 2018) and dried in an oven at 50°C for 48h. The dried samples were ground in a household grinder. The extraction process was performed by the soaking method as previously described (Harborne, 1980). Briefly, 100g of each dry powder was added in 1l of distilled water, and the mixture was kept in dark for 48h. After that, the concentrated liquid was centrifuged (10, 000 x *g* at 4°C, 15min; Sobye et al., 2018), and the resulting precipitate was discarded. Finally, the supernatant was then filtered using a 0.22μm syringe filter and used for preparing the desired prebiotic concentrations (2, 25, 50, 75, and 100%). In this assay, glucose was removed from all culture media (broth/agar) containing extracts as previously described (Hoseinifar et al., 2017).

### *In vitro* Evaluation of Prebiotics Properties of the Extracts

Each of the extracts from artichoke and mushroom was added to the glucose-free MRS medium at different concentrations (2, 25, 50, 75, and 100%). The MRS medium supplemented with glucose as a carbon source was maintained as a positive control group. The pH of the media was adjusted by 0.1N HCl to 5.8. The glucose-free MRS and MRS media containing each of the extracts (50ml) were inoculated with a culture of the probiotic strain La or Lb at a concentration of 10^7^ cells/ml. The solutions were incubated at 37°C for 48h under aerobic conditions after which the optical density (600nm) was measured as previously described (Hoseinifar et al., 2017).

### Diet Preparation

A commercial diet for small fishes, kindly provided by the Blue Line Company (Italy), was used as a basal diet and for the preparation of the experimental diets. The proximate composition of the diets is mentioned in Table 1. Based on the outcome from *in vitro* studies, we designed seven experimental diets. Diet 1: the basal diet was used as the control diet, Diet 2: the basal diet supplemented with 1% mushroom extract prepared from 50% concentrated extract, Diets 3 and 4: the basal diet supplemented with La at 10^7^ colony-forming units per gram of diet (CFUg^−1^) and Lb at 10^7^CFUg^−1^, respectively, Diet 5: the basal diet supplemented with La at 10^7^CFUg^−1^ and 1% of a mushroom extract prepared from 50% concentrated extract, Diet 6: the basal diet supplemented with Lb at 10^7^CFUg^−1^ and 1% mushroom extract prepared from a 50% concentrated extract, and Diet 7: the basal diet supplemented with La and Lb, each at 10^7^CFUg^−1^ and mushroom extract at 1% prepared from a 50% concentrated extract. The supplements were sprayed on the feed, mixed manually, and then each experimental diet was coated with 5% gelatin. To avoid the possible effects of gelatin, the control diet was also coated with 5% gelatin. All the diets were dried in a clean place at room temperature (25°C) and were then stored at 4°C until use. Diets were prepared weekly throughout the feeding trial and also analyzed for the viability of bacteria by culturing random samples of diets containing probiotics in MRS broth (Ashouri et al., 2018).

**Table 1:** Chemical composition of the basal diet.

Chemical composition	(%)
Dry matter	0.5
Crude protein	60
Crude lipid	17
Ash	10.5
Mineral premix	(%)
Na total	0.5
Ca total	1.5
P total	1.8
Vitamin premix	(per kg of feed)
A	1,000 Ul
D_3_	1,000 Ul
E	300mg/kg

### Experimental Animals and Design

Before initiation of the feeding trial, zebrafish larvae, obtained from a private sector farm in Gorgan, Golestan province, Iran, were kept for 2weeks in a holding tank for acclimatization during which they were fed with basal diet. The larvae were regularly monitored for their health and performance. No mortality was recorded during this period. After 2weeks, a group of 840 larvae with a mean initial weight and length of 75.9±1.0mg and 16.0±1.0mm, respectively, were randomly distributed into seven experimental groups (diets 1–7) with three replicates per group. Each group was maintained in an aquarium of 60L capacity containing 30L of water at a density of 40 fish per aquarium. Each diet was fed thrice daily (8:00, 12:30, and 18:00h) at 5% of the body weight for 120days (Lin et al., 2012; Hoseinifar et al., 2015). Round-the-clock aeration was provided to all the aquaria. Siphoning of uneaten feed and faecal matter was done daily. An approximately 25% of the aquarium water was removed daily and replaced with well-aerated freshwater. The experiment was conducted in the 14:10h light–dark cycles (Mohammadi Arani et al., 2019). Water temperature, pH, and dissolved oxygen were monitored daily and maintained approximately at 25±2°C, 7±0.2, and 7.9±0.1mgL^−1^, respectively (Safari et al., 2018). No mortality was recorded during the feeding period. The zebrafish larvae were kept and handled following the ethical guidelines for *in vivo* experiments developed by the Gorgan University of Agriculture and Natural Resources, Iran.

### Sample Collection and Growth Study

At the end of the feeding trial, fish in each tank fasted for 24h, and then 10 fish per aquarium (30 fish per treatment) were randomly sampled. They were immediately anesthetized using 200mgL^−1^ clove powder and their weight and length were measured at accuracy levels of 0.001g and 1mm, respectively (Mohammadi Arani et al., 2019). The growth and survival of the fish were monitored based on the following standard formula (Abedian-Amiri et al., 2017):


$$\mathrm{Weight gain}\;\left(\%\right)=\left({\mathrm{fW}}_{2}-W_{1}\right)\times 100/W_{1}.$$

$$\mathrm{Specific growth rate}\;\left(\%{\mathrm{day}}^{-1}\right)=\left({\mathrm{LnW}}_{2}-{\mathrm{LnW}}_{1}\right)\times 100/t.$$

$$\mathrm{Food conversion ratio}=\mathrm{feed intake}\;\left(\mathrm{mg}\right)/\mathrm{weight gain}\;\left(\mathrm{mg}\right).$$

$$\mathrm{Condition factor}=\mathrm{final weight}\;\left(\mathrm{mg}\right)\times 100/\mathrm{final length}\;{\left(\mathrm{mm}\right)}^{3}.$$

$$\begin{array}{l} \mathrm{Survival rate}\;\left(\%\right)=\mathrm{number of fish survived after}\;120\;\mathrm{days}\\ /\mathrm{initial number of fish stocked}\times 100. \end{array}$$

Where W_2_, W_1_, and *t* represent final weight (mg), initial weight (mg), and the trial period (day), respectively.

### Analysis of the Reproductive Performance

Ten fish from each experimental group were randomly sampled and sacrificed using an overdose of 500mgL^−1^ clove solution. The ovaries were completely dissected out and each of them was weighed with 0.0001mg precision. The number of ovules in each ovary was counted using a microscope. The reproductive performance was calculated following standard formulae (Mohammadi Arani et al., 2019) as listed below:


$$\begin{array}{l} \mathrm{Gonadosomatic index}=\mathrm{Gonad weight}\;\left(\mathrm{mg}\right)\times 100\\ /\mathrm{Final weight}\;\left(\mathrm{mg}\right). \end{array}$$

$$\begin{array}{l} \mathrm{Absolute fecundity}=\mathrm{Number of the ovule in ovary sample}\\ \times \mathrm{Weight of ovary}\;\left(\mathrm{mg}\right)/\mathrm{Weight of sample}\;\left(\mathrm{mg}\right). \end{array}$$

$$\begin{array}{l} \mathrm{Relative fecundity}=\mathrm{Total number of the ovule in the ovary}\\ \times \mathrm{Weight of fish}\;\left(\mathrm{mg}\right). \end{array}$$

Working fecundity=Number of hatched eggs;

$$\begin{array}{l} \mathrm{Hatching percentage}\;\left(\%\right)=\mathrm{Number of hatched eggs}\\ \times 100/\mathrm{Total number of eggs}. \end{array}$$

$$\begin{array}{l} \mathrm{Survival rate}\;\left(\%\right)=\mathrm{Number of larvae survived after}\;14\;\mathrm{days}\\ /\mathrm{Initial number of larvae}\times 100. \end{array}$$

### Analysis of *cyp19a* Gene

Nine fish (three fishes/replicates) were randomly sampled from each treatment. They were anesthetized using clove solution (500mgL^−1^), and then the ovaries were dissected out. Total RNA from each sampled ovary (100mg) was extracted using RNAx-PLUS extraction kit following the manufacturer’s instructions (Sinaclon, Iran). The samples were treated with RNAase-free DNase to remove DNA contamination. The quality and quantity of the isolated RNA were measured as described previously (Hosseini et al., 2016). The first-strand cDNAs were synthesized from total RNA using the cDNA synthesis kit (Genet Bio-Synthesis, South Korea) according to the manufacturer’s instructions. The RT-PCR reaction was run in triplicate using a standard protocol (Hosseini et al., 2016). *β-actin* was used as an internal control for gene expression normalization. The qPCR primers for *cyp19a* and *β-actin* were designed using Oligo7 as previously described (Mohammadi Arani et al., 2021; Table 2). To validate primers, qPCR efficiency was also taken into account for choosing the best qPCR primers pair with specific and correct size. The PCR efficiency and relative mRNA expression of *cyp19a* were calculated based on standard curve analysis with serial dilution of cDNA (including five dilutions) as previously described by Miandare et al. (2019).

**Table 2:** List of forward and reverse primers for qPCR analysis in zebrafish.

Gene	Primers	Sequence (5'-3')
*Cyp19a*	Forward	CCGTTCTTATGGCAGGTGAT
	Reverse	TTGTGTGGTCGATGGTGTCT
*β-actin*	Forward	GGTACCCATCTCCTGCTCCAA
	Reverse	GAGCGTGGCTACTCCTTCACC

Amplification and detection of specific products were performed using StepOne Real-Time PCR System (Life Technologies, Carlsbad, CA, United States), qPCR Master Mix containing SYBR® Green (Life Technologies, Carlsbad, CA, United States), and *cyp19a* specific primers (GenBank accession number AF183906). The samples were amplified in 25μl reaction mixtures containing 5.5μl of nuclease-free water, 1μl of each primer, 12.5μl of Maxima SYBR Green qPCR Master mix, and 5μl of cDNA template. The thermal cycle protocol was denaturation, annealing, and extension at 94°C for 20s, 60°C for 30s, and 72°C for 40s, respectively. The present sizes of all PCR products were verified by inspection of the dissociation curve and gel electrophoresis. The relative quantification of gene expression was calculated using the following equation (Livak and Schmittgen, 2001):


$$\mathrm{Relative gene expression}\;\left(2{-}^{\Delta \Delta \mathrm{ct}}\right)=2^{-\left(\Delta Ct\;\mathrm{sample}-\Delta Ct\;\mathrm{control}\right)}.$$

### Statistical Analysis

All the data were subjected to one-way ANOVA using statistical software Statistical Package for the Social Sciences (SPSS) version 16.0. Duncan’s multiple range tests were used to determine the differences among treatment means at *p*<0.05.

## Results

### Impact of Jerusalem Artichoke Extract on the Growth of *L. acidophilus*

**Figure 1:**
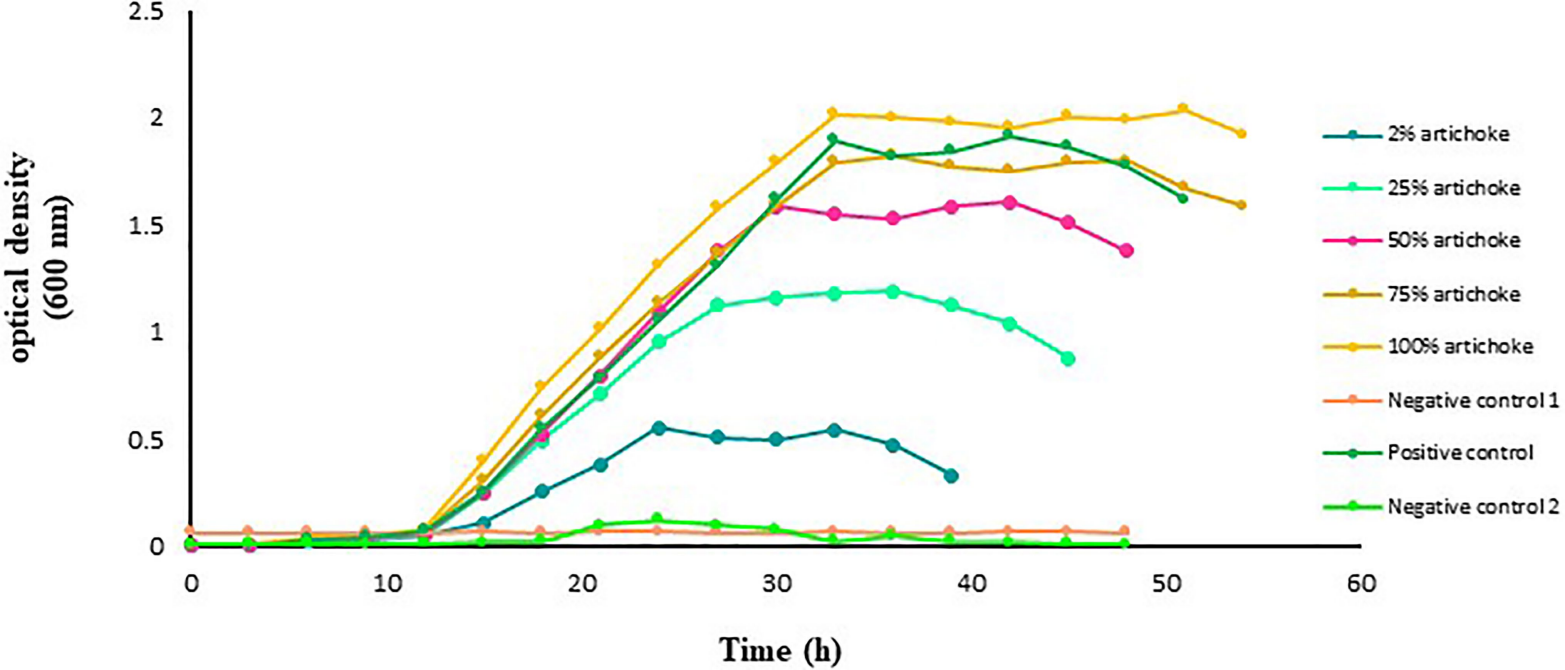
Growth of *Lactobacillus acidophilus* in De Man Rogosa Sharpe (MRS) broth medium with and without Jerusalem artichoke extract added at an increasing concentration of 2, 25, 50, 75, or 100%. *Lactobacillus acidophilus* grown in MRS broth medium with a standard amount of glucose served as a positive control. The glucose-free MRS medium with 100% of artichoke extract and without *L. acidophilus* served as negative control 1. The MRS medium free from both glucose and extract but with *L. acidophilus* served as negative control 2. The data points are the mean values of three replicates.

The growth rate of *L. acidophilus* in the function of time, cultured in the different experimental media was different (Figure 1). No growth was recorded in the glucose-free and artichoke extract-free culture medium. However, in the glucose-free MRS medium with artichoke extract as a substrate, a marked increase in the growth of *L. acidophilus* was recorded. Maximum improvement in the growth of *L. acidophilus* was noted in the medium that contained 100% artichoke extract. This was followed by those that were grown in the medium that contained 75% extract. The growth of *L. acidophilus* cultured in the medium with 2, 25, or 50% artichoke extract as substrate was lower than that in the medium that contained a standard amount of glucose (i.e., positive control).

### Impact of Mushroom Extract on the Growth of *L. acidophilus*

**Figure 2:**
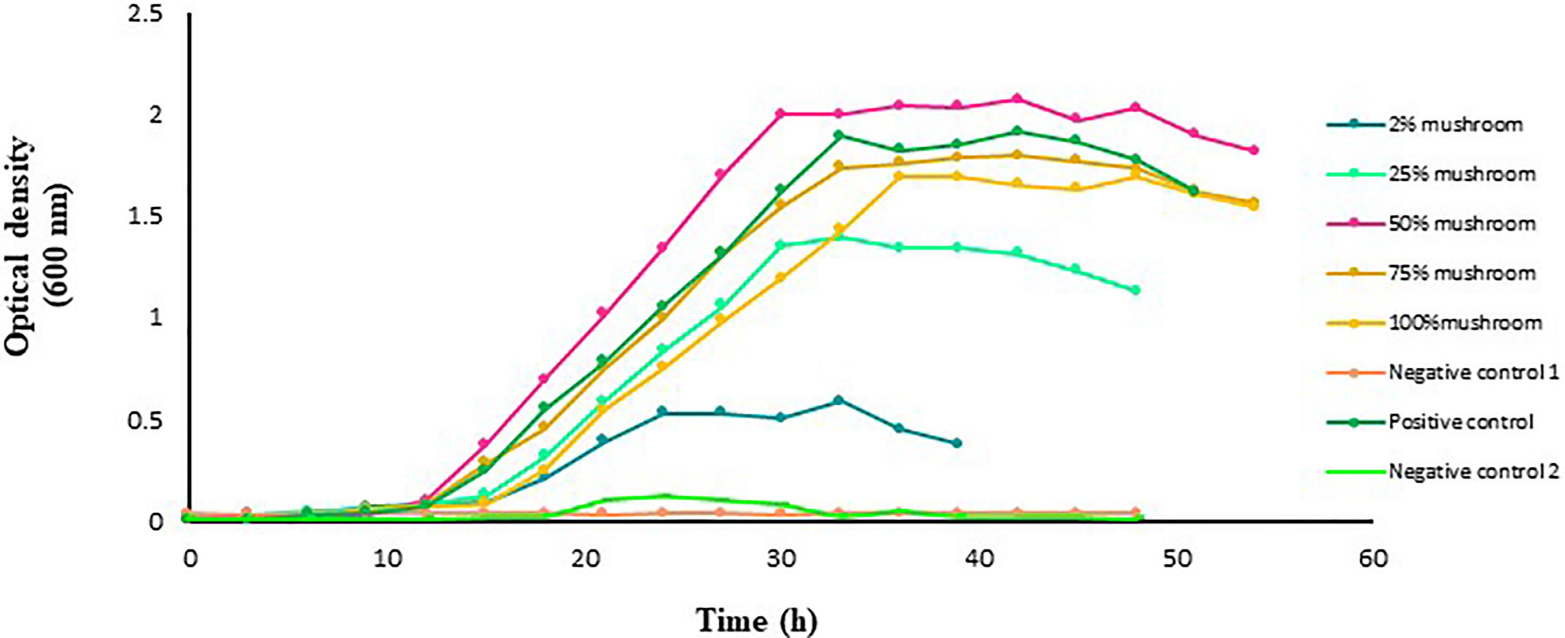
Growth of *L. acidophilus* in MRS broth medium with and without mushroom extract added at an increasing concentration of 2, 25, 50, 75, or 100%. *Lactobacillus acidophilus* grown in MRS broth medium with a standard amount of glucose served as a positive control. The glucose-free MRS medium with 100% of mushroom extract and without *L. acidophilus* served as negative control 1. The MRS medium free from both glucose and extract but with *L. acidophilus* served as negative control 2. The data points are the mean values of three replicates.

The growth of *L. acidophilus* was affected by the absence of glucose or mushroom extract in the medium (Figure 2). However, in the presence of mushroom extract, a marked improvement in the growth of *L. acidophilus* was observed, with the maximum being recorded in the medium that contained 50% extract. The growth of *L. acidophilus* cultured in the medium with 2, 25, or 100% mushroom extract as substrate was lower than that in the medium that contained a standard amount of glucose (i.e., positive control). *Lactobacillus acidophilus* cultured in the medium containing only glucose or 75% mushroom extract as substrate exhibited a similar growth rate.

### Impact of Jerusalem Artichoke Extract on the Growth of *L. delbrueckii Subsp. bulgaricus*

**Figure 3:**
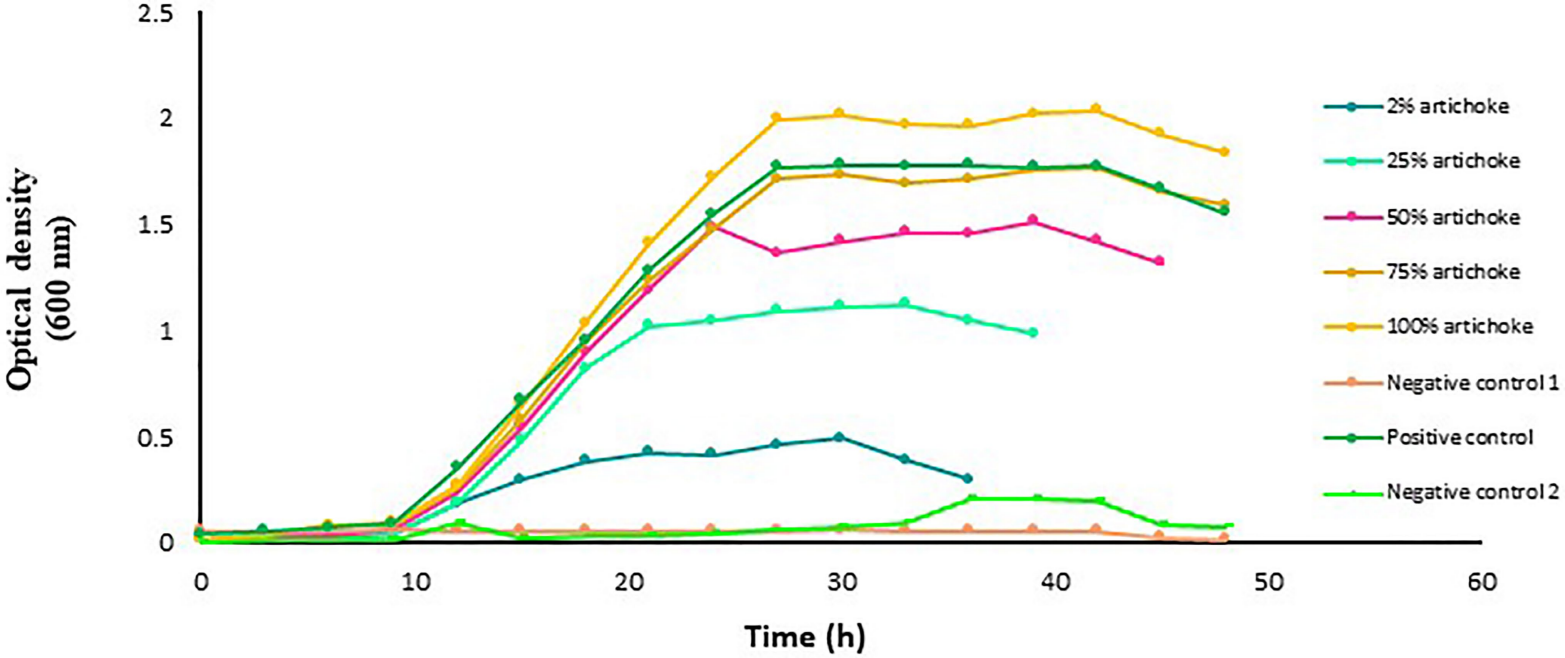
Growth of *Lactobacillus delbrueckii* subsp. *bulgaricus* in MRS broth medium with and without artichoke extract added at an increasing concentration of 2, 25, 50, 75, or 100%. *Lactobacillus acidophilus* grown in MRS broth medium with a standard amount of glucose served as a positive control. The glucose-free MRS medium with 100% of artichoke extract and without *L. acidophilus* served as negative control 1. The MRS medium free from both glucose and extract but with *L. acidophilus* served as negative control 2. The data points are the mean values of three replicates.

The growth of *L. delbrueckii* in the MRS culture medium was directly associated with the concentration of artichoke extract, which increased with the increase in the extract concentration (Figure 3). Maximum optical density reached by *L. delbrueckii* culture was in the medium that contained 100% artichoke extract. In the glucose-free MRS medium that contained artichoke extract at a concentration lower than 75%, the growth of *L. delbrueckii* in the function of time was relatively lower than that in the positive control.

### Impact of Mushroom Extract on the Growth of *L. delbrueckii Subsp. bulgaricus*

**Figure 4:**
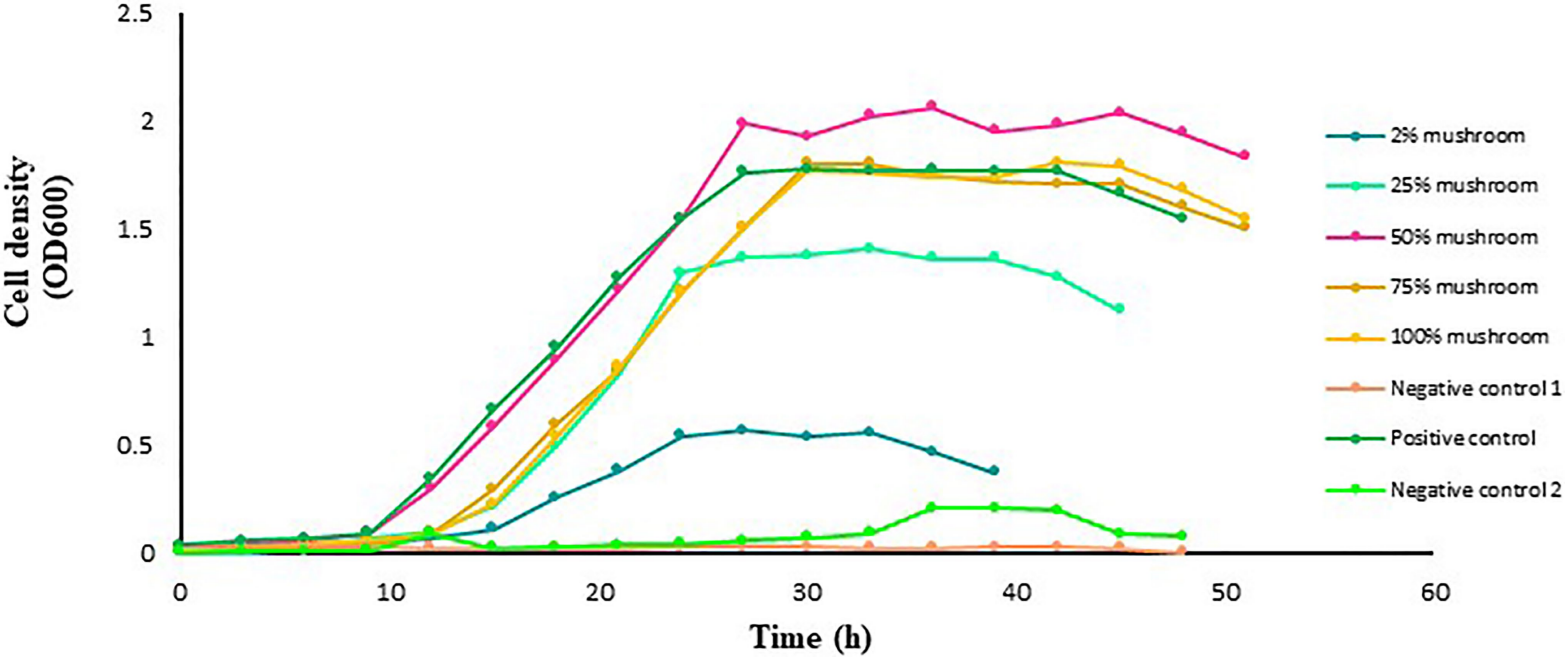
Growth of *L. delbrueckii* subsp. *bulgaricus* in MRS broth medium with and without mushroom extract added at an increasing concentration of 2, 25, 50, 75, or 100%. *Lactobacillus acidophilus* grown in MRS broth medium with a standard amount of glucose served as a positive control. The glucose-free MRS medium with 100% of mushroom extract and without *L. acidophilus* served as negative control 1. The MRS medium free from both glucose and extract but with *L. acidophilus* served as negative control 2. The data points are the mean values of three replicates.

The mushroom extract added at concentration of 50% in the culture medium markedly promotes the growth of *L. delbrueckii* in comparison to the controls (Figure 4). Increasing the extract concentration to 75% and higher did not further improve the growth of *L. delbrueckii*. In contrast, the growth exhibited a pattern similar to that in the positive control. The growth of *L. delbrueckii* cultured in the medium with 2 or 25% mushroom extract as substrate was lower than that in the positive control.

### Impact of the Plant Extracts on the Specific Growth Rate, Doubling Time of the *Lactobacillus* Probionts, and the pH of the Culture Media

**Figure 5:**
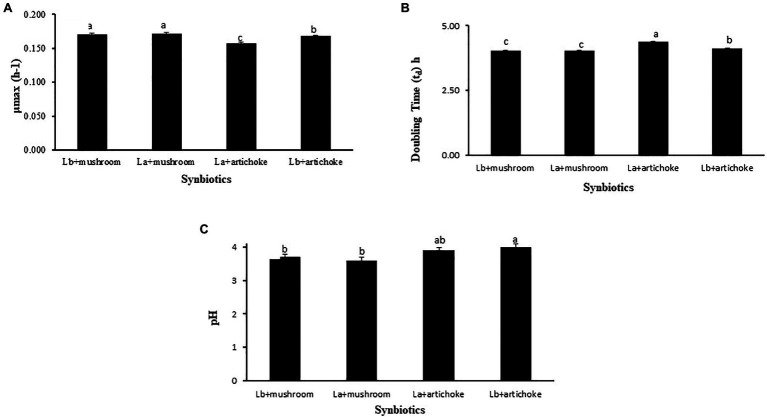
Comparison of the **(A)** specific growth rate (μmax; h^−1^), **(B)** doubling time (T_d_; h), and **(C)** pH of the medium of *L. acidophillus* with that of *L. delbrueckii* subsp. *bulgaricus* grown in MRS medium containing 50% mushroom or 100% artichoke extract. Bars with different letters indicate a significant difference (*p*<0.05). Data are presented as mean±SE.

A comparative analysis was carried out to determine the extracts that supported the best in promoting the growth indices, in terms of specific growth rate and doubling time, of the tested probiotic strains. The doses (50% of mushroom extract and 100% of artichoke extract) that showed the best performances in the bacterial growth studies were used for comparison purposes. As shown in Figure 5A, the specific growth rate of both the *Lactobacillus* probionts cultured in the medium with 50% mushroom extract was significantly (*p*<0.05) higher than those that were cultured with artichoke extract (100%) as substrate. No significant difference was observed in the doubling time between *L. acidophilus* and *L. delbrueckii* cultured on mushroom extract as substrate (*p*>0.05).

The doubling time of *L. acidophilus* and *L. delbrueckii* cultured in the medium containing 50% mushroom extract was significantly shorter in comparison to that of the probionts cultured on artichoke extract as substrate (*p*<0.05; Figure 5B). No significant difference was observed in the specific growth rate between *L. acidophilus* and *L. delbrueckii* cultured on mushroom extract as substrate (*p*>0.05).

The pH of the MRS medium containing a culture of *L. acidophilus* or *L. delbrueckii* in the presence of 50% mushroom extract was significantly lower than that of the medium containing *L. acidophilus* or *L. delbrueckii* culture in combination with 100% artichoke extract (Figure 5C).

### Growth Performances and Survival

The final weight of the fish after 4weeks of feeding and the resulting average fish weight gain during this experimental period were determined for all the treatments. At the start of the experiment, the fish in all the experimental groups weighed between 75 and 77mg and were not significantly different (Table 3). The weight of the fish in the experiment groups, irrespective of the treatment increased by 3–5-fold over the 4-week experimental period (Table 3). When calculating the growth variables, such as average fish weight gain and specific growth rate (SGR), no significant difference could be observed between the groups of fish fed a control diet, diet 3 supplemented with only La, and diet 5 supplemented with both La and mushroom extract. However, the group fed diet 6 supplemented with Lb and mushroom extract exhibited the highest weight gain and SGR values, and was significantly different from that of the control. Fish fed diet 7 supplemented with La, Lb, and mushroom extract in combination, their weight gain and SGR values did not differ significantly from that of the fish fed diet 6, however, was significantly higher than that of the control. The weight gain and SGR values in the groups fed diet 2 with mushroom extract as a supplement and diet 4 with Lb as a supplement were significantly higher than the control. However, the values were still significantly lower than the highest values that were observed for the diet 6 fed group. The average food conversion ratio (FCR) was 2.4 in the case of the control group. A similar FCR of 2.3 was found for zebrafish fed with diet 3 or diet 5. The feeding of diet 7 with Lb, La, and mushroom extract as a supplement resulted in a significant decrease in the FCR value. This was translated in a value of 1.8 for the diet 7 fed group, while the largest effect, although not significantly different from the latter, was noted for the fish fed diet 6 with an FCR value of 1.7. No mortality was recorded during the feeding period and the condition factor of the fish remained the same among the different experimental groups.

**Table 3:** Growth performance and survival of zebrafish fed different experimental diets for 120days.

Growth indices	Control diet	Diet 2	Diet 3	Diet 4	Diet 5	Diet 6	Diet 7
BW_i_ (mg)	77.4±1.1^a^	76.5±0.9^a^	75.6±1.0^a^	74.9±0.9^a^	76.7±0.5^a^	74.9±0.7^a^	75.3±1.0^a^
BW_f_ (mg)	265.8±5.4^c^	298.8±4.7^b^	269.4±2.5^c^	299.1±3.7^b^	269.0±5.9^c^	331.0±4.4^a^	327.7±5.7^a^
WG (%)	243.7±11.9^c^	290.8±10.6^b^	256.5±11.5^c^	299.6±14.0^b^	250.8±8.8^c^	342.0±12.7^a^	335.6±13.1^a^
SGR (% day^−1^)	1.03±0.03^c^	1.14±0.02^b^	1.06±0.00^c^	1.15±0.01^b^	1.05±0.02^c^	1.24±0.01^a^	1.23±0.02^a^
FCR	2.4±0.1^a^	2.1±0.1^b^	2.3±0.0^a^	2.0±0.0^b,c^	2.4±0.1^a^	1.7±0.0^d^	1.8±0.1^c,d^
K Factor (%)	0.51±0.02^a^	0.56±0.05^a^	0.53±0.02^a^	0.55±0.03^a^	0.52±0.03^a^	0.56±0.02^a^	0.54±0.03^a^
SR (%)	100.0±0.0^a^	100.0±0.0^a^	100.0±0.0^a^	100.0±0.0^a^	100.0±0.0^a^	100.0±0.0^a^	100.0±0.0^a^

Data are represented as mean *±* SE. Means with different superscript letters in each row represent significant differences (Duncan’s test, *p* <0.05). Ab, Agaricus bisporus; La, Lactobacillus acidophilus; and Lb, L. delbrueckii subsp. Bulgaricus, BW_i_, initial body weight; BW_f_, final body weight; WG, weight gain; SGR, specific growth rate; FCR, food conversion ratio; SR, survival rate; and K Factor, Fulton’s condition factor. Diet 1 (control diet), Diet 2 (supplemented with 1% mushroom extract prepared from 50% concentrated extract), Diet 3 (supplemented with La at 10^7^ CFUg^−1^ of diet), Diet 4 (supplemented with Lb at 10^7^ CFUg^−1^ of diet), Diet 5 (supplemented with La at 10^7^ CFUg^−1^ of diet +1% mushroom extract prepared from 50% concentrated extract), Diet 6 (supplemented with Lb at 10^7^ CFUg^−1^ of diet +1% mushroom extract prepared from 50% concentrated extract), and Diet 7 (supplemented with La and Lb, each at 10^7^ CFUg^−1^ of diet +1% mushroom extract prepared from 50% concentrated extract).

### Reproduction Performances

The results obtained for reproductive indices are shown in Table 4. As a general trend, the Gonadosomatic Index (GSI), absolute fecundity, and relative fecundity were influenced by the dietary supplements. No significant difference of the three indicated indices could be observed between the control groups, the group fed diet 3 supplemented with La, and the group fed diet 5 supplemented with both La and mushroom extract. However, the group fed diet 7 supplemented with a mixture of La, Lb, and mushroom extract exhibited the highest GSI, absolute fecundity, and relative fecundity values, and was significantly different from that of the control. The working fecundity of the fish fed different experimental diets did not differ significantly; neither did the hatching percentage of the fish in different experimental groups. The survival percentage of the fry after 4weeks of hatching in the different experimental groups was between 71 and 77% and was not significantly different.

**Table 4:** Reproduction performance and fry survival of zebrafish fed different experimental diets for 120days.

Reproductive indices	Control diet	Diet 2	Diet 3	Diet 4	Diet 5	Diet 6	Diet 7
GSI (%)	7.1±0.1^d^	7.5±0.1^c^	7.1±0.1^d^	7.4±0.1^c^	7.2±0.1^d^	7.7±0.1^b^	7.9±0.1^a^
AF	100.0±2.7^c^	104.0±3.4^a,b^	99.0±2.7^b^	104.0±3.2^a,b^	100.0±2.3^b^	105.0±5.0^a,b^	110.0±5.7^a^
RF	254.0±5.0^c^	256.0±7.0^c^	262.0±3.0^c,b^	271.0±4.0^a,b^	262.0±5.0^c,b^	275.0±3.0^a,b^	2,940±6.0^a^
WF	75.7±6.1^a^	73.0±5.5^a^	76.0±6.1^a^	75.0±6.4^a^	71.3±6.1^a^	75.0±5.5^a^	75.3±5.9^a^
HR (%)	83.0±1.9^a^	82.0±1.9^a^	84.0±1.2^a^	84.0±1.2^a^	84.0±1.5^a^	83.0±1.5^a^	84.0±2.0^a^
SR (%)	71.0±4.0^a^	75.0±6.0^a^	72.0±5.0^a^	72.0±4.0^a^	71.0±6.0^a^	75.0 ± 09.0^a^	77.0±7.0^a^

Data are represented as mean±SE. Means with different superscript letters in each row represent significant differences (Duncan’s test, *p* <0.05). Ab, Agaricus bisporus; La, Lactobacillus acidophilus; Lb, L. delbrueckii subsp. Bulgaricus; GSI, gonadosomatic index; AF, absolute fecundity; RF, relative fecundity; WF, working fecundity; HR, hatching percentage; and SR, fry survival rate. Diet 1 (control diet), Diet 2 (supplemented with 1% mushroom extract prepared from 50% concentrated extract), Diet 3 (supplemented with La at 10^7^ CFUg^−1^ of diet), Diet 4 (supplemented with Lb at 10^7^ CFUg^−1^ of diet), Diet 5 (supplemented with La at 10^7^ CFUg^−1^ of diet +1% mushroom extract prepared from 50% concentrated extract), Diet 6 (supplemented with Lb at 10^7^ CFUg^−1^ of diet +1% mushroom extract prepared from 50% concentrated extract), and Diet 7(supplemented with La and Lb, each at 10^7^ CFUg^−1^ of diet +1% mushroom extract prepared from 50% concentrated extract).

### Expression of *cyp19a* Gene

**Figure 6:**
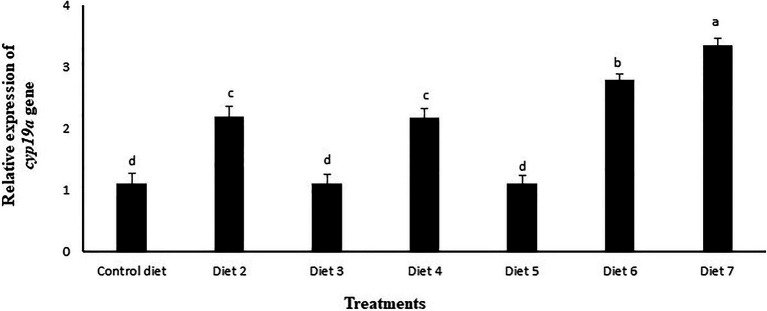
Effects of feeding various experimental diets for 120days on the relative expression of *cyp19a* gene in the ovary of zebrafish, normalized against *β-actin* factor as reference gene. Bars with different letters indicate significant differences (*p*<0.05). Data are presented as mean±SE of three replicates. Diet 1 (control diet), Diet 2 (supplemented with 1% mushroom extract prepared from 50% concentrated extract), Diet 3 (supplemented with La at 10^7^CFUg^−1^ of diet), Diet 4 (supplemented with Lb at 10^7^CFUg^−1^ of diet), Diet 5 (supplemented with La at 10^7^CFUg^−1^ of diet +1% mushroom extract prepared from 50% concentrated extract), Diet 6 (supplemented with Lb at 10^7^CFUg^−1^ of diet +1% mushroom extract prepared from 50% concentrated extract), and Diet 7(supplemented with La and Lb, each at 10^7^CFUg^−1^ of diet +1% mushroom extract prepared from 50% concentrated extract).

The relative abundance of mRNA encoding for the *cyp19a* gene was determined by qPCR in the ovaries of female zebrafish fed different experimental diets for 120days. At the end of the feeling trial, it could be observed that in the group fed with diet 3 supplemented with La, and in the group fed diet 5 supplemented with both La and mushroom extract, no significant upregulation of the *cyp19a* gene was recorded relative to the control. However, the groups fed diet 2, diet 3, diet 6, or diet 7; all these groups exhibited a significant increase in the expression level of *cyp19a* gene. Among all the experimental groups, those that were fed diet 7 supplemented with a mixture of La, Lb and mushroom extract recorded the highest expression level of *cyp19a* gene (*p*<0.05; Figure 6).

## Discussion

Here, we tested the compatibility of two extracts from the plant Jerusalem artichokes and button mushrooms with two different *Lactobacillus* probiotics to develop a synbiotic formulation with the aim to improve the growth and reproductive performances of farmed fishes. The two plant extracts were selected in this study because of their prebiotic potential as demonstrated in previous studies on farmed fishes (for details, see, Amiri et al., 2018; Boonanuntanasarn et al., 2018; Hoseinifar et al., 2019b; Sewaka et al., 2019; Abedalhammed et al., 2020). It is well-established that prebiotics can selectively promote the growth of probiotics. The underlying mechanism behind this growth promotion is the enzymatic hydrolysis of the non-digestible polysaccharides, followed by the uptake of the hydrolysis products, or even the direct uptake of small oligomers (Huynh et al., 2017; Nunpan et al., 2019; Massa et al., 2020). Thus, one of the prerequisites of a synbiotic should be the ability of the probiotic bacteria to ferment the prebiotic components. This ability of the probiotic bacteria is highly specific and is not only species and strain-dependent but also is markedly influenced by the dose, composition, and origin of the prebiotic (de Melo Pereira et al., 2018; Zoumpopoulou et al., 2018). Initially, we employed *in vitro* approach to monitor the growth of the probiotic lactobacilli in the presence of the different doses of the plant-based prebiotics, with the aim to select interesting combination(s) for further verification under *in vivo* conditions. Our results showed that when the two probiotic strains were tested in the presence of the two different plant extracts, a clear increase in the growth rate was achieved by both the strains, however, the growth promotion effect of the plant extracts on the tested probiotic strains was dose-dependent. In the case of the artichoke extract, it was the dose of 100% that resulted in higher growth rates of the *L. acidophilus* and *L. delbrueckii* (see, Figures 1, 3) but in the case of the mushroom extract, it was at the dose of 50% where the probiotic strain exhibited maximum growth rate (Figures 2, 4). The observed increase in the growth rate of the probionts in response to the addition of artichoke extract in the medium could be attributed to the presence of inulin-type fructans, such as inulin, fructooligosaccharide, in the artichoke extract (Yaseen, 2018; Rattanakiat et al., 2020; Zhu et al., 2020). Fructans are one of the most known prebiotics that were shown to stimulate probiotics growth in several organisms, including fish (Li et al., 2019; Manikandan et al., 2020; Ghafarifarsani et al., 2021; Zimmermann et al., 2021). In an *in vitro* study conducted by Van Doan et al. (2015), it was noted that Jerusalem artichoke extract at 5% concentration caused maximum growth in the bacterial population of *Lactobacillus plantarum*, finding that is in line with that of ours. Similarly, the observed increase in the growth of each of the two probiotics cultured together with mushroom extract could be attributed to the prebiotic components, such as chitin, hemicellulose, β- and α-glucans, mannans, xylans, and galatians, which are widely available in mushrooms (Mirończuk-Chodakowska and Witkowska, 2020; Atila et al., 2021). The findings from our study are supported by several other studies. For instance, Sawangwan et al. (2018) showed that the ability of edible mushroom extract as a prebiotic had a positive effect on the growth of probiotics *L. acidophilus*. This finding was complemented by other studies that showed that extract of oyster mushroom, *Pleurotus eryngii*, could promote the growth of not only *Lactobacillus* spp. but also other important probiotics species, such as *Bifidobacterium* spp., and *Enterococcus faecium* (Synytsya et al., 2009; Van Doan et al., 2016). It is interesting to mention that the artichoke and mushroom extracts at a concentration lower than 75% or mushroom extract at a higher concentration of 100% did not result in improvement in the growth-promotion of the probiotic species under study with respect to the corresponding species cultured in the presence of glucose. Reduced bacterial growth at lower and higher concentrations may be related to unavailability of suitable nutrients and unoptimized pH or high levels of beta-glucan in the culture medium, which has a negative effect on the bacterial growth; therefore, the optimization of nutrient concentration is very important (Reischke et al., 2015; Meshram et al., 2016; Rojo-Cebreros et al., 2017). As beta-glucan is made up of glucose monomers, when taken with probiotics, the bonds between them are broken down by beta-gluconase enzymes. Also, on the other hand, high levels of glucose have inhibitory properties for bacterial growth; the reduction in bacterial growth at a concentration of 100% of the mushroom extract can be justified (Reischke et al., 2015).

Our results showed that both the probiotic species showed a preference for 50% mushroom extract as a source of prebiotics since their growth with mushroom extract resulted in higher growth rates than those obtained in the presence of 100% artichoke extract (Figure 5). There is evidence suggesting that different prebiotic compounds display a varying degree of fermentability and short-chain fatty production in the presence of a probiotic strain (Carlson et al., 2018; Wang et al., 2019). It has been described that prebiotic compounds with higher polymerization degree are relatively more difficult to ferment than similar prebiotics with lower one (Mueller et al., 2016; Langa et al., 2019; Méndez et al., 2019). The preference of the two probiotics under study for the mushroom extract, over the artichoke extract, could be explained by the variation in the composition of the prebiotic compounds between artichoke and mushroom extracts, with the latter being more fermentable. A significant decline of pH in the medium that contained mushroom extract and each of the two probiotics relative to that in the medium that contained artichoke extract and the probiotics supports our above explanation that mushroom extract was better fermented by the tested probiotics.

Having observed that the mushroom extract at a concentration of 50% caused the most prominent growth-promoting effect, when cultured with each of the tested probiotic species response, we, therefore, selected the indicated dose of the mushroom extract and combined it with La and/or Lb to develop synbiotics. We then validate their beneficial effects using the *in vivo* model organism, zebrafish focusing on the survival, growth, and reproductive performances of zebrafish. Feeding of a diet supplemented with Lb and mushroom extract (diet 6) in combination caused a positive influence on the weight gain percentage and SGR of zebrafish. During the rearing periods of 120days, the average fish weight gain percentage increased in the group fed diet 6 increased to 342 and 335%, respectively, relative to 243% for the control group. Accordingly, the group fed diet 6 and diet 7; both showed an increase in the SGR by a fold of 1.2. This resulted in an FCR that was significantly lower for the group fed a diet supplemented with Lb and mushroom extract (diet 6) or with La, Lb, and mushroom extract (diet 7) in combination. Our results also showed that the condition factor (K), which is an indicator of the fish general condition, remained within the optimum range during the entire experimental period, and this was reflected in the survival results as no significant mortality was observed due to feeding experimental diets. This result suggests that the selected *Lactobacillus* species and the mushroom extract (50%) either alone or in combination caused no harmful effect on the fish, at least in our described experimental condition, and hence can be considered safe for use in fish feed. The underlying rationale for the observed improvement in the growth of zebrafish in response to feeding the developed synbiotic-supplemented diet could be multifactorial. For instance, (i) improvement in the gut microbial flora, (ii) inhibition of the attachment and cloning of pathogenic microorganisms to the intestinal wall of the fish, (iii) and maintenance of beneficial microbial population in the gut, (iv) improvement in the feed intake and digestion, (v) production of enzymes, vitamins, and other beneficial substances for the host, and (vi) improvement in the immune status of the fish (Aftabgard et al., 2018; Ashouri et al., 2018; Mirghaed et al., 2018; Dawood et al., 2020; Kazuń et al., 2020; Yang et al., 2020; El-Nobi et al., 2021; Mohammadi Arani et al., 2021; Zhandalgarova et al., 2021). However, at present, this is mere speculation and warrants further research. To our knowledge, no report is available on the use of probiotics La or Lb in combination with mushroom extract as a synbiotic formulation compound in farmed fishes. However, the beneficial effects of the synbiotic formulation on zebrafish performance parameters including weight gain percentage, SGR FCR, and survival are in agreement with previous studies (Safari et al., 2018; Mohammadian et al., 2019; Priya et al., 2021). For example, a similar improvement in the growth performances was observed in a study with a commercial synbiotic that comprised of a probiotic *Entercoccus faecium* and fructooligosaccharide as a prebiotic in zebrafish (Nekoubin et al., 2012b).

The reproductive performances of organisms, including fish, are gated by the state of energy reserves in the organism, and that there is a balance between energy homeostasis and fertility (Gioacchini et al., 2010; Mohammadi Arani et al., 2021). The impact of energy status on the reproductive axis is conveyed through several neuroendocrine hormones and metabolic cues, such as kiss1, kiss2, leptin, and Cyp19a, whose nature and mechanisms of action have begun to be deciphered in fish (Carnevali et al., 2016; Doering et al., 2019; Mohammadi Arani et al., 2021; Shan et al., 2021). Cyp19a is the key steroidogenic enzyme that plays an important role in the conversion of androgens to estrogens in the sexual differentiation and reproductive cycles of vertebrates (Zheng et al., 2019; Doering et al., 2021). Over the past few years, evidence has been put forward to demonstrate that microbial supplements can improve the reproductive performances of fishes (for details see, Ahmadifard et al., 2019; Gabriel, 2019; Mohammadi Arani et al., 2021). For instance, in a study carried out on zebrafish, feeding of the probiotic *Lactobacillus rhamnosus* caused a significant effect on follicle development, production of ovulated eggs, and hatching rate (Gioacchini et al., 2010). In another study, zebrafish fed a diet supplemented with probiotic *Pediococcus acidilactici* exhibited a significant improvement in the gonado somatic index, fecundity, and hatching percentage. Interestingly, this increase in the reproductive performances of the fish was associated with a significant increase in the expression of the *cyp19a* gene (Mohammadi Arani et al., 2021). Similar results have also been obtained with a synbiotic product Biomin imbo, which improved several reproductive indices like fecundity, hatching rate, hatching time, and the rate of germinal vesicle breakdown in zebrafish (Nekoubin et al., 2012b) and fecundity in angelfish *Pterophyllum scalare* (Nekoubin et al., 2012a). In agreement with the above findings, our results also demonstrated a stimulatory role of the synbiotic comprising of La, Lb, and mushroom extract in combination (diet 7) on a set of key reproductive indices of zebrafish. It is worth highlighting that despite the improvement in the reproductive performances, the hatching percentage, and the survival rate of the fry were not influenced by the feeding of synbiotic. A possible explanation for the observed response could be attributed to the culture conditions that were maintained at the optimum level for the culture of the fish. Consistent with our findings, Rodriguez-Estrada et al. (2009) observed no significant effect of feeding rainbow trout for 12weeks with *Enterococcus faecalis* and MOS/PHB on the survival rate of the fish. Also, similar results were observed in Japanese flounder (*Paralichthys olivaceus*) when fed with *Bacillus clausii* and MOS/FOS (Ye et al., 2011).

The molecular mechanisms behind the stimulating effects of prebiotics, probiotics, and/or synbiotics on the reproductive performances in organisms are not yet fully known. However, in our study, we found a significant upregulation of the *cyp19a* gene in the ovaries of the fish fed synbiotic-supplemented diet, and this coincided well with the improvement of the reproductive indices. The stimulatory role of synbiotic on female zebrafish reproductive performances may be due to both the activation of the endocrine control described above and to the direct action of synbiotic-mediated *cyp19a* on the ovary. In this study, although the *cyp19a* gene induced by synbiotic appeared to improve the reproductive factors, the induction of other metabolic signals, such as kiss1, kiss2, and leptin by this product and their collective involvement in governing the reproduction process cannot be excluded. Further studies are required to substantiate this assumption by developing or using primers specific for these genes of interest.

In essence, the *in vitro* screening assay in the broth showed a preference of *L. acidophilus* and *L. bulgaricus* for mushroom extract as a prebiotic. A synbiotic formulation, developed with the selected combination of *L. acidophilus*, *L. bulgaricus*, and 50% mushroom extract, showed a positive influence on the growth and reproductive performances of the *in vivo model* organism zebrafish. Our findings also imply that the improvement in the reproductive indices was associated with the upregulation of a *cyp19a* gene. Overall results suggest that a combination of *L. acidophilus*, *L. bulgaricus*, and mushroom extract can be considered as a potential synbiotic for aquaculture species. Further research is warranted to unravel the specific mechanisms involved in this synbiotic effect and the validation of this formulation following nutritional trials in aquaculture species of economic importance.

## Data Availability Statement

The raw data supporting the conclusions of this article will be made available by the authors, without undue reservation.

## Ethics Statement

The animal study was conducted following the ethical guidelines set by the Gorgan University of Agriculture Sciences and Natural Resources, Iran.

## Author Contributions

HZ conducted the experiment and executed most of the data processing and analysis. MS, SH, and HP participated in the designing of the experiments and data analysis and guided and supervised the work. HZ and KB wrote the bulk of the manuscript and critically edited by MS, SH, and HP. All authors contributed to the article and approved the submitted version.

## Funding

This work was supported by the institutional PhD grant provided by the Gorgan University of Agriculture and Natural Resources, Iran.

## Conflict of Interest

The authors declare that the research was conducted in the absence of any commercial or financial relationships that could be construed as a potential conflict of interest.

## Publisher’s Note

All claims expressed in this article are solely those of the authors and do not necessarily represent those of their affiliated organizations, or those of the publisher, the editors and the reviewers. Any product that may be evaluated in this article, or claim that may be made by its manufacturer, is not guaranteed or endorsed by the publisher.
